# Gefitinib for non-small-cell lung cancer patients with epidermal growth factor receptor gene mutations screened by peptide nucleic acid-locked nucleic acid PCR clamp

**DOI:** 10.1038/sj.bjc.6603466

**Published:** 2006-11-14

**Authors:** A Sutani, Y Nagai, K Udagawa, Y Uchida, N Koyama, Y Murayama, T Tanaka, H Miyazawa, M Nagata, M Kanazawa, K Hagiwara, K Kobayashi

**Affiliations:** 1 Department of Respiratory Medicine, Saitama Medical University, 38, Morohongo, Moroyama-machi, Iruma-gun, Saitama 350-0495, Japan

**Keywords:** lung neoplasms, EGFR, gefitinib, tyrosine kinase inhibitor, clinical trial, PNA-LNA PCR clamp

## Abstract

This study was prospectively designed to evaluate a phase II study of gefitinib for non-small-cell lung cancer (NSCLC) patients with epidermal growth factor receptor (EGFR) mutations. Clinical samples were tested for EGFR mutations by peptide nucleic acid-locked nucleic acid PCR clamp, and patients having EGFR mutations were given gefitinib 250 mg daily as the second treatment after chemotherapy. Poor PS patients omitted chemotherapy. Of 107 consecutive patients enrolled, samples from 100 patients were informative, and EGFR mutations were observed in 38 patients. Gefitinib was given to 27 patients with EGFR mutations, and the response rate was 78% (one complete response and 20 partial responses; 95% confidence interval: 58–93%). Median time to progression and median survival time (MST) from gefitinib treatment were 9.4 and 15.4 months, respectively. Grade 3 hepatic toxicity and skin toxicity were observed in one patient each. There were significant differences between EGFR mutations and wild-type patients in response rates (78 *vs* 14%, *P*=0.0017), and MST (15.4 *vs* 11.1 months, *P*=0.0135). A Cox proportional hazards model indicated that negative EGFR mutation was a secondary prognostic factor (hazards ratio: 2.259, *P*=0.036). This research showed the need for screening for EGFR mutations in NSCLC patients.

Gefitinib is an orally active epidermal growth factor receptor (EGFR) tyrosine kinase inhibitor that competes with ATP for the ATP-binding site in the cytoplasmic tail of EGFR (Brehmer *et al*, 2005). Gefitinib was studied in two trials: the Iressa® Dose Evaluation in Advanced Lung Cancer (IDEAL)-1 and IDEAL-2 trials (Fukuoka *et al*, 2003; Kris *et al*, 2003). Patients enrolled in the IDEAL-1 and IDEAL-2 trials were required to have failed only one prior platinum-containing regimen, and a platinum plus docetaxel, respectively. In the IDEAL trials, the response rates ranged from 9 to 19%. Grades 3 and 4 toxicities were relatively uncommon. Based on the IDEAL trials, gefitinib received registration approval by the US Food and Drug Administration (FDA) for the second- and third-line treatment of non-small-cell lung cancer (NSCLC) (Siegel-Lakhai *et al*, 2005). The Iressa® Survival Evaluation in Lung Cancer (ISEL) trial investigated gefitinib in second- and third-line NSCLC patients to investigate the survival benefit of gefitinib monotherapy compared with placebo. A total of 1692 patients who were refractory to or could not tolerate chemotherapy were enrolled. The results showed significantly greater tumour shrinkage in the gefitinib arm, but the overall survival durations were similar in both arms: 5.6 months in treated patients *vs* 5.1 months in patients received placebo. This failure of gefitinib to show a survival advantage over placebo resulted in controversy about the registration (Thatcher *et al*, 2005; Twombly, 2005).

In 2004, it was shown that mutations in the *EGFR* gene are significantly associated with response to two tyrosine kinase inhibitors, gefitinib (Lynch *et al*, 2004; Paez *et al*, 2004). The majority of EGFR tyrosine kinase domain mutations occur in two ‘hot spots’, exons 19 and 21. In exon 19, deletions eliminate four highly conserved amino acids (LREA). In exon 21, a missense point mutation substitutes an amino acid at position 858 (L858R). Among various mutations found in the EGFR tyrosine kinase domain, only the following have so far been positively associated with a response to gefitinib or erlotinib from retrospective analyses: G719C (exon 18), some of the common exon 19 deletions (LREA), L861Q (exon 21) and L858R (exon 21) (Pao and Miller, 2005). All such mutations result in conformational changes that lead to increased sensitivity to tyrosine kinase inhibitors.

Several retrospective studies have shown that higher rates of these mutations were found in females, in never-smokers, in Asians and in patients with adenocarcinomas (Mitsudomi *et al*, 2005; Tokumo *et al*, 2005). And a better response to gefitinib has been reported in patients harbouring EGFR mutations (Taron *et al*, 2005). These results indicate that screening of patients for EGFR tyrosine kinase domain mutations before treatment with gefitinib or other EGFR inhibitors may predict the clinical benefit of the treatment. However, approaches frequently required biopsy or surgical specimens, as well as skilful techniques (Lynch *et al*, 2004; Paez *et al*, 2004; Mitsudomi *et al*, 2005; Pao and Miller, 2005; Tokumo *et al*, 2005; Twombly, 2005). We developed a method, peptide nucleic acid-locked nucleic acid (PNA-LNA) PCR clamp, capable of detecting EGFR mutations in the presence of 100-fold background levels of wild-type EGFR from normal cells (Nagai *et al*, 2005). Because of its high sensitivity and specificity, PNA-LNA PCR clamp was considered suitable to detect EGFR mutations both in histological samples such as surgical specimens, and in cytological samples such as sputum and pleural effusions.

This phase II study was prospectively designed to evaluate the effect of gefitinib in NSCLC patients with *EGFR* gene mutations screened by PNA-LNA PCR clamp.

## PATIENTS AND METHODS

The two-step protocol of this phase II study, that is (i) testing for EGFR mutations by PNA-LNA PCR clamp, and (ii) administering gefitinib to NSCLC patients with EGFR mutations, were approved by the Institutional Review Board (IRB) of Saitama Medical University Hospital. This study was performed in accordance with the Declaration of Helsinki (1964, amended in 2000) of the World Medical Association.

### Primary entry criteria and testing for EGFR mutations

Consecutive NSCLC patients who were admitted in our single institution and gave written informed consent for testing for *EGFR* gene mutations by PNA-LNA PCR clamp, which was designed to detect 11 different EGFR mutations. Detection rate (sensitivity) by PNA-LNA PCR clamp is 89% and its accuracy (specificity) is 100%. In PNA-LNA PCR clamp, existence of other types of EGFR mutation is realised by seeing escape of the inhibition of amplification by the clamp primer, and, in this case, a direct sequencing method is employed to seek other types of EGFR mutation. Finally, overall sensitivity and specificity of this system is 97 and 100%, respectively, using clinical samples (submitting). The cytology specimens were divided into pathology samples (the main sample) and PNA-LNA PCR clamp samples (a small aliquot). When the pathologist confirmed a pathology sample to contain cancer cells (i.e. rated as classes IV or V), the cells in the PNA-LNA PCR clamp samples, which had been collected and stored in the AL buffer (a buffer containing protein denaturant: Qiagen, Hilden Germany), were then subjected to the analysis. While, the paraffin-embedded tissue specimens were serially thin-sliced: one slice was used to confirm the presence of cancer cells under microscopy, whereas the others were investigated by the PNA-LNA PCR reaction.

The PNA-LNA PCR clamp method has been described in detail (Nagai *et al*, 2005). Briefly, primers used were

F18: 5′-GGTAGCTGTTCAGTTAAAGAACACC-3′ and
B18: 5′-CCTTTGGTCTGTGAATTGGTC-3′ for exon 18,
F19: 5′-CTGGATGAAATGATCCACACG-3′ and
B19: 5′-TGGGTAGATGCCAGTAATTGC-3′ for exon 19, and
F21: 5′-CTGGATGGAGAAAAGTTAATGGTC-3′ and
B21: 5′-CAGCAAGTACCGTTCCCAAAG-3′ for exon 21.

PCR primers were designed manually or by using Primer 3 software (http://frodo.wi.mit.edu/cgi-bin/primer3/primer3.cgi) so that the *T*_m_ values were between 55 and 60°C. Fluorogenic probes containing LNA were manually designed and confirmed by the LNA *T*_m_ prediction tool (http://lna-tm.com/) to have *T*_m_ values between 54 and 56°C. Peptide nucleic acid clamp primers, 14- to 18- mer in length, were designed according to the guidelines (Ugozzoli *et al*, 2004). LNA-containing oligos were synthesised by IDT (Coralville, IA, USA), and PNA oligos were synthesised by Greiner Japan, Tokyo, Japan. For PNA-LNA PCR clamp, PCR primers (200 nM each), fluorogenic probes (100 nM each) and a PNA clamp primer (5 *μ*M) were added to the Basic Mixture containing 25 mM TAPS pH 9.3, 50 mM KCl, 2 mM MgCl_2_, 1 mM 2-mercaptoethanol, 200 *μ*M each of dNTPs and 1.25 U of Takara Ex Taq HS (Takara Bio, Shiga, Japan). For PCR reactions, PCR and the real-time amplification monitoring for the PNA-LNA PCR clamp were performed using Smart Cycler II (Cepheid Sunnyvale, CA, USA). PCR cycling was a 30-s hold at 95°C followed by 45 cycles of 95°C for 3 s and 62°C (exons 18 and 19) or 56°C (exon 21) for 30 s.

### Secondary entry criteria and treatment schedule

After testing for EGFR mutations by PNA-LNA PCR clamp, patients who satisfied the following inclusion criteria were enrolled: (a) EGFR mutations, (b) inoperable stage III–IV and recurrence after operation, (c) measurable region(s), (d) adequate bone marrow (white blood cell count⩾4000 mm^−3^; platelet count⩾100 000 mm^−3^; hemoglobin⩾9.5 g dl^−1^), total bilirubin ⩽1.5 mg dl^−1^, transaminases less than twice the upper limit of normal, and serum creatinine level ⩽1.5 mg dl^−1^, (e) age 20 years, (f) no medical problems severe enough to prevent compliance with the study requirements, and (g) secondary informed consent to be treated by gefitinib.

Gefitinib (250 mg p.o. daily) was given as the second treatment after disease was on progression by cytotoxic chemotherapy for PS 0–2 patients with EGFR mutations. In the case of poorer PS owing to advanced disease, the first line chemotherapy was omitted and gefitinib was administered as the first therapy. The other patients not enrolled into the phase II study were clinically treated by appropriate therapies according to our institutional manual, and their data on EGFR mutation status and survival time were collected and analysed.

### Evaluation

Patients were evaluated by physical examination, chest X-ray, bone scintiscan, computed tomography (CT) of the head, chest and abdomen, and fiberoptic bronchoscopy, and clinical stages were then determined according to the tumour-node-metastasis system. Chest X-rays were assessed at least every 2 weeks after the initial evaluation, and a chest CT was planned to evaluate tumour response and tumour progression. Tumour response was classified in accordance with Response Evaluation Criteria in Solid Tumours.

Before the first course, each patient was subjected to a complete blood cell count (CBC), serum chemistry for renal and hepatic functions, electrolyte analysis and urinalysis. CBC, serum chemistry, electrolyte analysis and urinalysis were assessed at least once a week after the initial evaluation. The NCI Common Toxicity Criteria (version 3) was used to grade organ system damage.

### Statistical analysis

The primary end point of this study was the response to gefitinib for patients with EGFR mutations. Sample size was determined to be 25 patients with *EGFR* gene mutations. We chose a 75% response rate as a desirable target level and a 50% response rate as uninteresting. Our design had a power in excess of 90% and less than 10% type I error. A total number of patients to be tested by PNA-LNA PCR clamp was decided to be more than 100 patients because about 30% of Japanese NSCLC patients were reported to have EGFR mutations in previous articles (Mitsudomi *et al*, 2005; Tokumo *et al*, 2005).

Secondary end points were survival, side effects and clinical usefulness of PNA-LNA PCR clamp. Differences in response to gefitinib and survival after gefitinib therapy between patients with wild *EGFR* genes and those with mutant *EGFR* genes were assessed to indicate a clinical usefulness of screening by PNA-LNA PCR clamp. Furthermore, differences in overall survival from the initial treatment between the groups, and whether EGFR mutations were a prognostic factor were also investigated. Survival curves were drawn using the Kaplan–Meier method, and Logrank was calculated for evaluating survival differences between the groups. A Cox proportional hazards model (multiple variate) using EGFR mutations, sex, stage and PS was also employed using the data from all the patients enrolled by the primary entry criteria. All the analyses were calculated by SPSS® 11.0J.

## RESULTS

**Figure 1:**
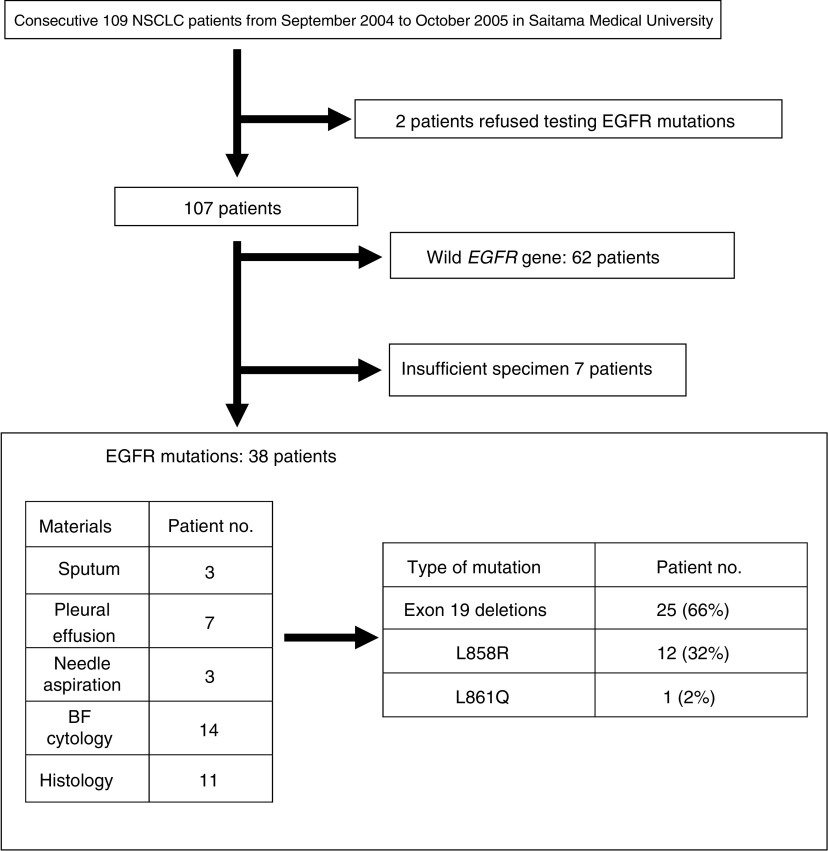
Patients entered and source of specimen and type of EGFR mutation.

From September 2004 to October 2005, samples from 107 Japanese NSCLC patients were tested by PNA-LNA PCR clamp but two patients refused consent for checking for EGFR mutations (Figure 1). One hundred patients (93%) of the 107 patients provided adequate samples for evaluation of EGFR mutation status, and samples from seven patients did not provide enough DNA. PNA-LNA PCR clamp detected EGFR mutations in 38 patients (38%; 95% confidence interval (CI): 28–48%) who were 15 male and 23 female patients (Table 1). Their median age was 62 years old, and, of the 38 patients, 33 patients had adenocarcinoma. Exon 19 deletions, L858R and L861Q were found in 25 (66%), 12 (32%) and 1 (2%) patients, respectively (Figure 1). On the other hand, 62 patients (51 men/11 women; median age: 66 years; adenocarcinoma: 43 patients) were judged to have wild-type EGFR. There were significant differences between EGFR mutation-positive and EGFR mutation-negative groups with regard to sex (male *vs* female: *P*=0.00001), histology (adenocarcinoma *vs* non-adenocarcinoma: *P*=0.02) and smoking (>20 pack-years *vs* <20 pack-years: *P*=0.003) (Table 1).

### Phase II study

Of the 38 patients with EGFR mutations, gefitinib was given to 27 patients. The other 11 patients were not treated by gefitinib because they did not meet the secondary entry criteria.

Four patients and 23 patients were given gefitinib as the first-line and the second-line treatment, respectively. All of the 27 patients were assessed for response. One patient showed a complete response (CR) and 20 patients showed partial responses (PRs). The overall response rate was 78% (95% CI: 62–94%) (Table 2). The response rate in the 23 patients treated by gefitinib after chemotherapy was 74% (95% CI: 56–92%). When patients were stratified by EGFR mutation types, response rates were 75% (15 out of 20 patients) for exon 19 deletions, and 86% (six out of seven patients) for L858R. There were no significant differences in the response rates between the mutation types (*χ*^2^ test: *P*=0.557).

**Figure 2:**
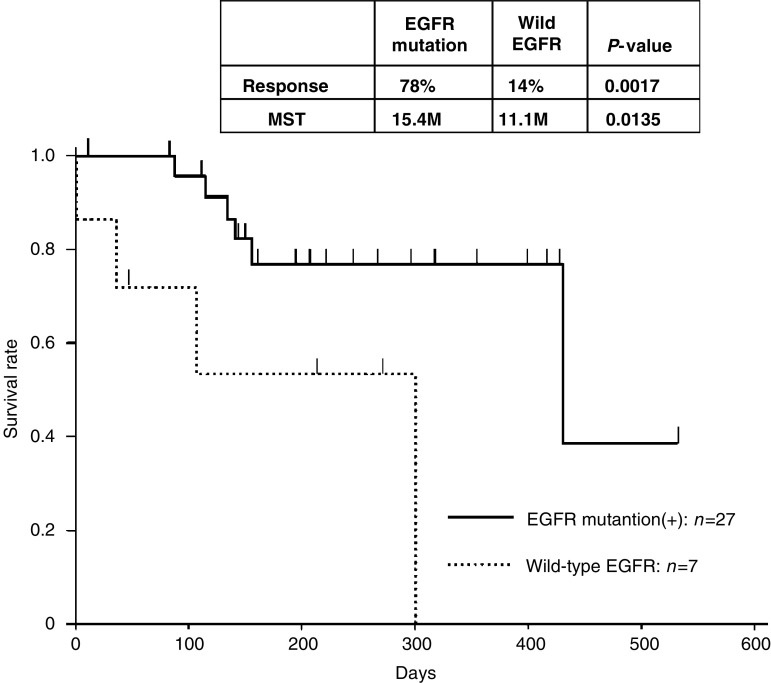
Survival time curves *after gefitinib treatment* in patients with and without EGFR mutation are shown.

For the 27 patients, median time to progression (TTP) from the gefitinib treatment was 9.4 months. And median survival time (MST) from the gefitinib treatment was 15.4 months (Figure 2). There were also no significant differences in survival time after the gefitinib treatment between the patients with exon 19 deletions and L858R (Kaplan–Meier, logrank: *P*<0.455). The 21 patients with CR/PR had a longer TTP and overall survival (14.4+ and 19.1+ months, respectively) than patients with stable disease/progression (3.1 and 5.6 months, respectively).

All 27 eligible patients were assessable for toxicity (Table 3). Grade 3 drug-related hepatic toxicity was observed in one patient (4%), and Grade 3 skin toxicity occurred in one patient (4%). Other gastrointestinal toxicities were mild and acceptable. No lung toxicities were observed.

### Clinical usefulness of PNA-LNA PCR clamp

To investigate the clinical usefulness of PNA-LNA PCR clamp screening, patients with EGFR mutations detected by the test were compared to those with wild EGFR. The response rates were significantly different between patients with EGFR mutations (78%) and patients with wild EGFR (14%) (*χ*^2^ test, *P*=0.0017, Figure 2). Median survival time after the gefitinib treatment was significantly different between patients with EGFR mutations (15.4 months) and those with wild-type EGFR (11.1 months) (Kaplan–Meier, logrank: *P*=0.0135, Figure 2).

**Figure 3:**
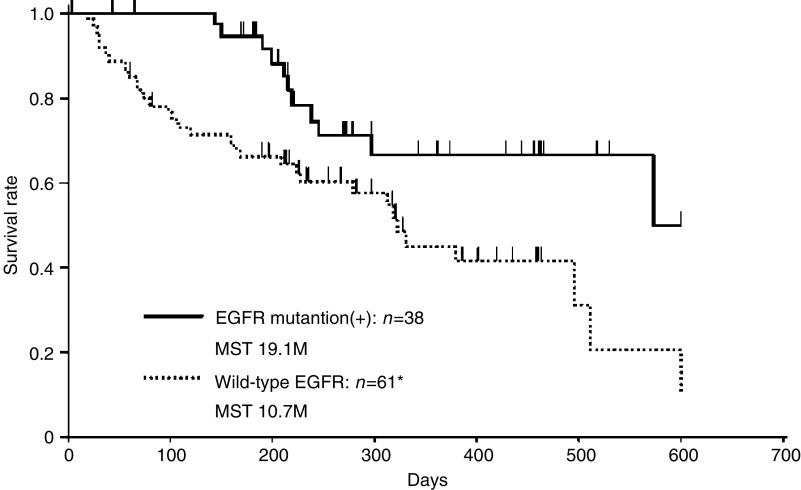
Overall survival curves *after the initial treatments* in patients with and without EGFR mutation are shown.

Furthermore, to clarify whether EGFR mutation status tested by PNA-LNA PCR clamp could be a prognostic factor for NSCLC patients, the relationship between EGFR mutation status and overall survival were evaluated using 99 patients except for one patient who was lost in follow-up. Figure 3 shows the comparison of overall survival after the initial treatments between NSCLC patients with EGFR mutations and those with wild-type EGFR by the Kaplan–Meier method. Overall survival after the initial treatment was significantly different between the groups (EGFR mutations: 19.1 months and wild-type EGFR: 10.7 months, logrank: *P*<0.0108). The Cox proportional hazards model (multiple variate) was also applied using EGFR mutations, sex, stage and PS. The latter three factors are well known as prognostic factors in NSCLC patients (Brundage *et al*, 2002). The Cox proportional hazards model indicated that detecting EGFR mutations was a secondary prognostic factor (Table 4).

## DISCUSSION

With PNA-LNA PCR clamp, we were able to determine EGFR mutation status in a majority of the NSCLC patients using clinical samples such as sputum and BF cytology. To determine EGFR mutations, direct sequencing or PCR-single strand conformational polymorphism methods are frequently employed (Lynch *et al*, 2004; Paez *et al*, 2004; Mitsudomi *et al*, 2005; Pao and Miller, 2005; Tokumo *et al*, 2005; Twombly, 2005). However, these methods are time-consuming and require specimens that consist mostly of cancer cells. Another approach that analysis of an increased *EGFR* gene copy number, based on fluorescence *in situ* hybridisation analysis, could be used as a predictive marker for sensitivity to gefitinib (Bell *et al*, 2005; Hirsch and Witta, 2005; Takano *et al*, 2005). However, this method also needs specimens consisting mostly of cancer cells, significant operation time and skilful technicians who have intertechnician variability. Thus, these methods can be employed only at some academic medical centres. The preferred and practical method is one that can sensitively, specifically and quickly detect EGFR mutations from specimens used for the diagnosis of lung cancers without removing contaminating normal cells. Peptide nucleic acid-locked nucleic acid PCR clamp can rapidly (within 2 hours) detect EGFR mutations from all specimens used to diagnose lung cancers, that is, sputum, pleural effusion and bronchial washing which contain many normal cells. This method is able to sensitively and specifically detect all 11 types of EGFR mutations (Nagai *et al*, 2005) in the presence of 100-fold wild-type EGFR background levels. These 11 mutations account for more than 95% of EGFR mutations found (Lynch *et al*, 2004; Twombly, 2005).

PNA-LNA PCR clamp prospectively detected EGFR mutations in 38% (95% CI: 28–48%) of the consecutive patients with NSCLC. Patients who were EGFR mutation-positive were mostly women (61%) and had adenocarcinomas (87%), and significantly lower smoking index (34%). These results were consistent with the results of previous retrospective reports (Mitsudomi *et al*, 2005; Tokumo *et al*, 2005). Some clinical studies are trying to select patients to gefitinib treatment by clinicopathologic features of adenocarcinoma and non-smoker without testing EGFR mutations. Our data indicate such an approach is not feasible. For example, when selecting patients with adenocarcinoma and smoking >20 pack-years, 15 of the 38 patients with EGFR mutations (39%) would be missed, whereas 13 of the 62 patients without EGFR mutations (21%) would be mistakenly included.

Furthermore, the presence of EGFR mutations detected by the PNA-LNA PCR clamp was found to be a prognostic factor in Japanese patients with NSCLC in this prospective screening. A Cox proportional hazards model indicated that detecting EGFR mutations was a significant prognostic factor and was superior to sex or stage, indicating that incorporating the PNA-LNA PCR clamp into clinical studies and clinical practice is critical.

This phase II study clearly showed the favourable response to gefinitib in NSCLC patients with EGFR mutations. The response rate was 78% and the lower limit of the 95% confidential interval of response was 62%. In contrast to previous retrospective analyses (Riely *et al*, 2006; Hirsch *et al*, 2006), patients with exon 19 deletions were equally responsive compared to those with L858R in this study. This might be due to our small sample size, so these data need to be confirmed in a larger trial. In EGFR mutation-positive patients treated by gefitinib, TTP (9.4 months) of after the gefitinib treatment and MST (19.1 months) after the initial treatment were longer than in patients treated with the regimens using platinum doublet. Detection of EGFR mutations clearly differentiates gefinitib-sensitive patients from gefinitib-insensitive patients with regard to response rate and survival times.

Four patients with EGFR mutations received gefitinib as the first line treatment because they could not be given chemotherapy owing to poor PS. Two patients had meningitis carcinomatosa. One had multiple brain metastases. And one had repeated aspiration pneumonia owing to recurrent nerve palsy. All of these patients showed PR and obtained better PS. Their survival times were 190, 183+, 278+ and 296+ days, respectively, and all returned home. This experience taught us the usefulness of testing for EGFR mutations in patients with poor PS owing to advanced disease. Thus, even in Europe and the US where frequencies of EGFR mutations are low, incorporating testing for EGFR mutations in clinical practice may provide a huge benefit to some patients.

In conclusion, our study prospectively demonstrated the clinical benefit of gefitinib given to NSCLC patients with good PS as the second-line treatment harbouring EGFR mutations, and, also, gefitinib given to NSCLC patients with poor PS as the first-line treatment showed a favourable response. To attain this benefit, screening clinical samples at the time of diagnosis is imperative, and PNA-LNA PCR clamp is a good method to achieve this aim.

## Figures and Tables

**Table 1:** Patients' characteristics

	**Mutation**	**Wild-type**	
	**(*n*=38)**	**(*n*=62)**	***P*-value**
Male/female	15/23 pts	51/11 pts	0.00001
Median age (years) (s.d., range)	62 (10.0, 44–79)	66 (12.0, 32–81)	0.09
			
*Stage*			
I	1 pt (2.6%)	3 pts (4.8%)	0.175
II	1 pt (2.6%)	2 pts (3.2%)	
III	9 pts (23.7%)	23 pts (37.1%)	
IV	23 pts (60.5%)	22 pts (35.5%)	
Relapse	4 pts (10.5%)	12 pts (19.4%)	
			
*Histology*			
Adenocarcinoma	33 pts (86.8%)	43 pts (69.4%)	0.02
Squamous cell carcinoma	2 pts (5.3%)	12 pts (19.4%)	
Adenosquamous	1 pt (2.6%)	1 pt (1.6%)	
Large cell carcinoma	0 pt (0.0%)	1 pt (1.6%)	
Undifferentiated	2 pts (5.3%)	5 pts (8.1%)	
			
*Smoking*			
>20 pack-years	13 pts (34.2%)	40 pts (64.5)	0.003
			
ECOG PS			
0–2	34 pts (89.5%)	55 pts (88.7%)	0.906
3–4	4 pts (10.5%)	7 pts (11.3%)	
			
*Treatments* a			
Operation	10 pts (26.3%)	23 pts (37.1%)	0.948
Chemotherapy	30 pts (78.9%)	43 pts (69.4%)	
Irradiation	2 pts (5.3%)	6 pts (9.7%)	

ECOG PS=Eastern co-operative oncology group performance status; Pts=patients.

a All the treatments which were given to patients for the intervening periods of the diseases.

**Table 2:** Efficacy of gefitinib in patients with EGFR mutation

	**CR**	**PR**	**s.d.**	**PD**	**Response**
Prior chemotherapy (+)	1	16	5	1	17 pts/23 pts (74%)
					(95% CI: 56–92%)
Prior chemotherapy (−)	0	4	0	0	4 pts/4 pts (100%)
					
Exon 19 deletions	1	14	4	1	15 pts/20 pts (75%)
L858R	0	6	1	0	6 pts/7 pts (86%)
					
Total	1	20	5	1	21 pts/27 pts (78%)
					(95% CI: 62–94%)

CI=confidence interval; CR=complete response; EGFR=epidermal growth factor receptor; PD=progressive disease; PR=partial response; Pts=patients; s.d.= standard deviation.

**Table 3:** Side effects of gefitinib in patients with EGFR mutation

	**No. of patients with CTC grade (*n*=27)**		
	**2**	**3**	**4**
*Haematologic toxicity*			
Neutropenia	1	0	0
Thrombocytopenia	0	0	0
Anaemia	0	0	0
			
*Other toxicities*			
Diarrhoea	5	0	0
Nausea and vomiting	2	0	0
Acne/acneform	9	1	—
Abnormal liver function (AST, ALT)	1	1	0
Abnormal renal function	0	0	0
Acute lung injury	0	0	0

ALT=alanine aminotransferase; AST=aspartate aminotransferase; CTC=common toxicity criteria; EGFR=epidermal growth factor receptor.

**Table 4:** Cox proportional hazards analysis

	**Hazards ratio**	***P*-value**
Mutation	2.259	0.036
Performance status	1.542	0.002
Male/female	1.053	0.887
Stage	1.029	0.319

A Cox proportional hazards model (multiple variate) using EGFR mutations, sex, stage and PS was employed using the data from all the patients (*n*=99^*^) enrolled by the primary entry criteria.

^*^Data missing: one patient.
